# Subversion of actin dynamics by EspM effectors of attaching and effacing bacterial pathogens

**DOI:** 10.1111/j.1462-5822.2008.01136.x

**Published:** 2008-03-26

**Authors:** Ana Arbeloa, Richard R Bulgin, Georgina MacKenzie, Robert K Shaw, Mark J Pallen, Valerie F Crepin, Cedric N Berger, Gad Frankel

**Affiliations:** 1 Division of Cell and Molecular Biology, Imperial College London London SW7 2AZ, UK; 2 Division of Immunity and Infection, School of Medicine, University of Birmingham Birmingham B15 2TT, UK

## Abstract

Rho GTPases are common targets of bacterial toxins and type III secretion system effectors. IpgB1 and IpgB2 of *Shigella* and Map of enteropathogenic (EPEC) and enterohemorrhagic (EHEC) *Escherichia coli* were recently grouped together on the basis that they share a conserved WxxxE motif. In this study, we characterized six WxxxE effectors from attaching and effacing pathogens: TrcA and EspM1 of EPEC strain B171, EspM1 and EspM2 of EHEC strain Sakai and EspM2 and EspM3 of *Citrobacter rodentium*. We show that EspM2 triggers formation of global parallel stress fibres, TrcA and EspM1 induce formation of localized parallel stress fibres and EspM3 triggers formation of localized radial stress fibres. Using EspM2 and EspM3 as model effectors, we report that while substituting the conserved Trp with Ala abolished activity, conservative Trp to Tyr or Glu to Asp substitutions did not affect stress-fibre formation. We show, using dominant negative constructs and chemical inhibitors, that the activity of EspM2 and EspM3 is RhoA and ROCK-dependent. Using Rhotekin pull-downs, we have shown that EspM2 and EspM3 activate RhoA; translocation of EspM2 and EspM3 triggered phosphorylation of cofilin. These results suggest that the EspM effectors modulate actin dynamics by activating the RhoA signalling pathway.

## Introduction

Colonization, invasion and transversion of mucosal surfaces are alternative strategies used by pathogenic bacteria to cause infectious diseases. Many Gram-negative bacteria employ a type III secretion system (T3SS) to inject effector proteins into the cytosol of their mammalian host (reviewed in Galan and Wolf-Watz, 2006), which facilitate their unique infection strategies. T3SS effectors are targeted to different subcellular compartments and affect diverse signalling pathways and physiological processes. For example, enteropathogenic (EPEC) and enterohemorrhagic (EHEC) *Escherichia coli* belong to a family of medically important extracellular diarrhoegenic pathogens (reviewed in Kaper *et al.*, 2004) that colonize the gut mucosa by the attaching and effacing (A/E) mechanism (Knutton *et al.*, 1987). EHEC and EPEC inject dozens of T3SS effector proteins (Tobe *et al.*, 2006), including EspG and EspG2 which disrupt the microtubule network which may lead to formation of stress fibres at late stages of the infection via an indirect activation of RhoA (Matsuzawa *et al.*, 2004; Shaw *et al.*, 2005), Map which induces transient formation of filopodia (Kenny and Jepson, 2000), Tir which triggers extensive localized actin polymerization leading to formation of pedestal-shaped structures under adherent bacteria (Kenny *et al.*, 1997) and TccP/EspF_U_ which connects Tir to the actin cytoskeleton (Campellone *et al.*, 2004; Garmendia *et al.*, 2004).

The actin cytoskeleton (reviewed in Cossart and Sansonetti, 2004) and Rho GTPases (reviewed in Jaffe and Hall, 2005) are prominent targets of T3SS effectors (Finlay, 2005). There are 22 Rho GTPases in man which regulate various cellular processes, including actin polymerization, microtubule dynamics and cell cycle, morphogenesis and migration (reviewed in Jaffe and Hall, 2005). GTPases act as molecular switches cycling between GTP-bound (active) and GDP-bound (inactive) conformations. Switching a GTPase on and off is mediated by guanine nucleotide exchange factors (GEFs) and GTPase-activating proteins (GAPs) respectively. The Rho GTPases transmit signals in a GTP-dependent manner by activating and/or recruiting downstream effector proteins to their sites of action (reviewed in Jaffe and Hall, 2005).

Recently, Alto *et al.* (2006) assembled several known T3SS effectors into a single family that share the common motif Trp-xxx-Glu (WxxxE). This family includes the *Salmonella* effectors SifB and SifA, which is involved in intracellular survival and replication by maintaining the integrity of the *Salmonella*-containing vacuole (Beuzon *et al.*, 2000), the *Shigella* effectors IpgB2 and IpgB1, which is involved in bacterial cell invasion (Ohya *et al.*, 2005), the EPEC and EHEC effector Map (Kenny and Jepson, 2000) and the EPEC protein TrcA (bfpT-regulated chaperone-like protein gene) (Tobe *et al.*, 1999; Alto *et al.*, 2006). Although sharing no sequence homology with the Rho GTPases, Alto *et al.* (2006) suggested that IpgB1, IpgB2 and Map mimic, in a GTP-independent mechanism, the activated form of Rac-1, RhoA and Cdc42 respectively. It was shown that ectopic expression of IpgB2 induces formation of new stress fibres, while IpgB2^W62A^ and IpgB2^E66A^ were not biologically active; stress-fibre formation was RhoA-independent as IpgB2 was functional in the presence of RhoA inhibitors (e.g. C3 botulinum toxin) or dominant negative RhoA^T19N^. In addition, IpgB2 was shown to directly stimulate the activity of Rho-associated kinase (ROCK) and to interact with mDIA, two downstream effectors of RhoA.

The completed and ongoing genome projects of the A/E pathogens EHEC O157:H7 (strains Sakai and EDL933), EPEC O127:H6 (strain E2348/69), EPEC O111:NM (strain B171) and the mouse pathogen *Citrobacter rodentium* (strain ICC168) have revealed that the number of T3SS effector proteins encoded by this family has been greatly underestimated. A comprehensive study of the T3SS repertoire of EHEC O157:H7 has shown that among the dozens of T3SS effectors are two new putative members of the WxxxE proteins, EspM1 and EspM2 (Tobe *et al.*, 2006). Using Map and IpgB2 as index genes, we identified additional putative WxxxE effectors in the A/E pathogens, including EspM1 in EPEC B171 and EspM2 and EspM3 in *C. rodentium*. The aim of this study was to investigate the function and mechanism of cell signalling triggered by the A/E pathogen WxxxE effectors.

## Results

### Identification of novel WxxxE effectors in the A/E pathogen group

**Fig. 1:**
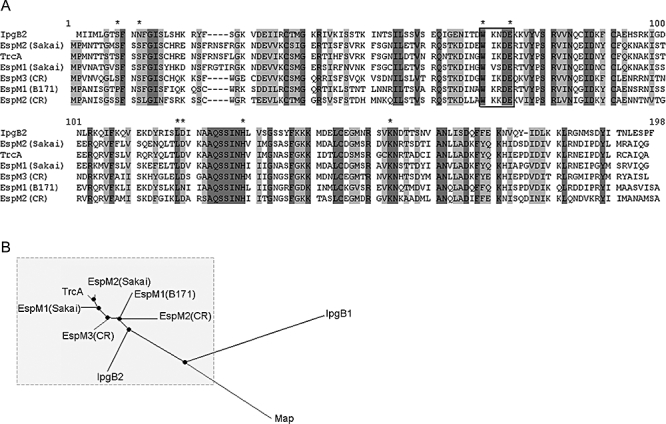
The EspM WxxxE effector proteins in A/E pathogen group. A. Multiple sequence alignment with hierarchical clustering of *S. exneri* IpgB2, EspM1 and EspM2 from EHEC O157:H7 Sakai, EspM2 and EspM3 from *C. rodentium* (CR) and TrcA and EspM1 from EPEC B171. Similar and identical residues are highlighted in bright and dark grey respectively. The conserved motif WxxxE is boxed. Conserved residues subjected to mutagenesis in this study are tagged with a star. B. Radial phylogeny tree showing the subfamily of IpgB2-like WxxxE effectors in the A/E group, EPEC E2348/69 Map and *Shigella* IpgB1 and IpgB2.

EspM1 and EspM2 of EHEC O157 are putative WxxxE effectors (Tobe *et al.*, 2006), sharing 76% identity (Fig. 1). EspM1 and EspM2 are closely related to the *Shigella* WxxxE effector IpgB2 (40% and 41% identity respectively) while sharing only 23% and 22% identity, respectively, with Map of EPEC E2348/69 (Fig. 1).

In order to define the repertoire of WxxxE proteins in EPEC strains E2348/69 and B171 and *C. rodentium*, we searched their genomes using the blast algorithm with IpgB2 and Map as index proteins. In EPEC B171, one homologue, TrcA, had already been identified (Tobe *et al.*, 1999; Alto *et al.*, 2006). Here we report an additional putative WxxxE effector, EspM1 (Accession #: AM910623) (Fig. 1), which is closely related to proteins encoded in the LEE of rabbit EPEC and EHEC O103:H2. We demonstrated that HA-tagged TrcA and EspM1 (B171) are translocated in a T3SS-dependent mechanism (data not shown). TrcA and EspM1 (B171) exhibit 41% and 46% identity, respectively, with IpgB2 (Fig. 1). Importantly, the blast searches revealed that EPEC E2348/69 harbours no other functional WxxxE effector than Map.

Searching the *C. rodentium* ICC168 genome sequence, we identified two putative WxxxE effectors, EspM2 (Accession #: AM910622) and EspM3 (Accession #: AM910621), which share 42% and 47% identity, respectively, with IpgB2. Using a TEM-1-β-lactamase fusion assay (Charpentier and Oswald, 2004), we demonstrated that EspM2 (CR) and EspM3 are translocated in a T3SS-dependent mechanism (data not shown). blast hits that we did not follow up include one open reading frame (ROD40861) that has a frame shift deletion upstream of the WxxxE motif and a distantly related IpgB2 homologue.

A radial phylogenetic tree, created based upon multiple sequence alignment with hierarchical clustering (Corpet, 1988), showed that all the IpgB2-like A/E effectors indeed cluster with IpgB2 and are distant from IpgB1 and Map (Fig. 1B). The nomenclature of the A/E WxxxE effectors was based on functional homology rather than sequence identity (see below).

### The WxxxE A/E effectors trigger formation of three distinct actin stress fibre architectures

**Fig. 2:**
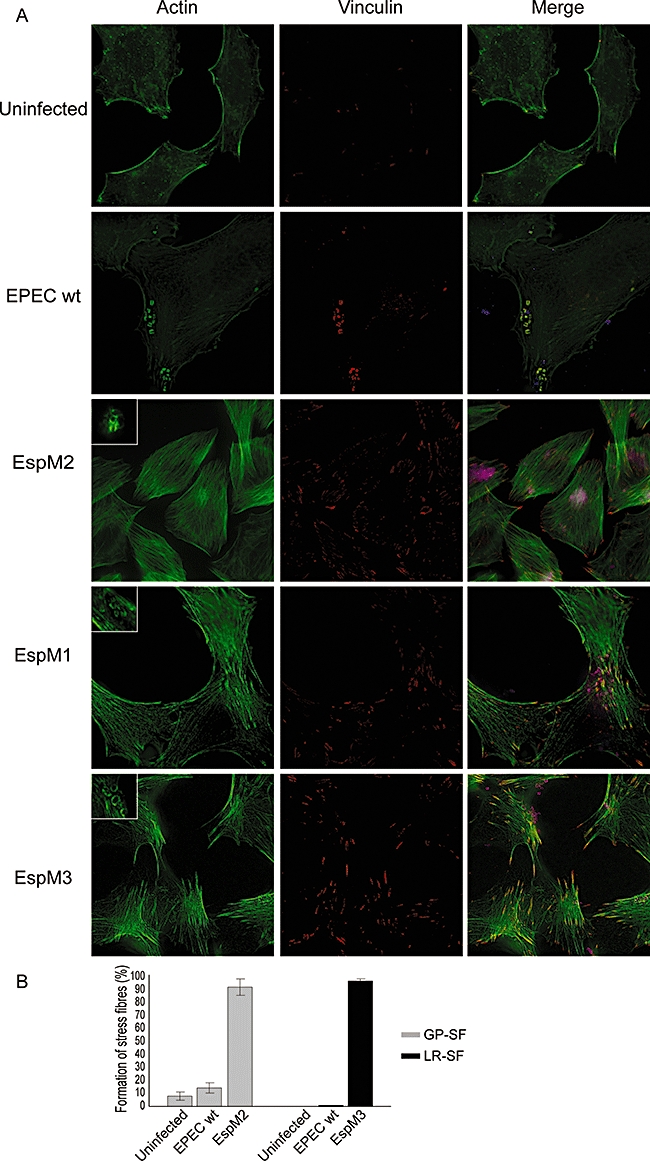
The EspM A/E effectors trigger formation of actin stress fibres in infected Swiss 3T3 cells. A. Fluorescence microscopy of uninfected Swiss 3T3 cell or cells infected for 90 min with wild-type E2348/69 and E2348/69 expressing EspM2 (Sakai), EspM1 (B171) and EspM3 (CR). Actin was stained with Oregon green phalloidin, EPEC were detected with rabbit anti-0127 antibody (merged column) and vinculin was visualised using a monoclonal mouse antibody. GP-SF, LP-SF and LR-SF were observed in cell infected with EPEC expressing EspM2, EspM1 and EspM3 respectively. All the recombinant strains also induced formation of actin pedestals (insets). B. Quantification of parallel and radial stress-fibre formation in uninfected Swiss cells or after 90 min infection with wild-type E2348/69 and E2348/69 expressing EspM2 and EspM3. One hundred cells were counted in triplicate in three independent experiments. Results are presented as mean ± SEM.

In order to determine if similarly to IpgB2, the EspM1, EspM2, TrcA and EspM3 homologues can remodel actin in the mammalian host cells, the genes were cloned into the expression vector pSA10 (Schlosser-Silverman *et al.*, 2000) and expressed in wild-type EPEC E2348/69 (lacking any of the IpgB2 homologues). Recombinant E2348/69 were used to infect serum-starved Swiss 3T3 cells, which generally lack stress fibres (Fig. 2) and are capable of dynamic actin signalling. Control infections with wild-type EPEC E2348/69 (Fig. 2) or EPEC E2348/69 carrying the empty pSA10 vector (results not shown) trigger efficient Tir-dependent actin-rich pedestals, while stress fibres were seen only in a small percentage of infected Swiss 3T3 cells (Fig. 2). In contrast, infection of Swiss 3T3 cells with E2348/69 expressing EspM2 (Sakai) (Fig. 2) and EspM2 (CR) (data not shown) induced simultaneous formation of actin pedestals and global parallel stress fibres (GP-SF), which were on different focal planes. These stress fibres were observed in *c*. 90% of cells after 1.5 h infection (Fig. 2). Applying anti-HA antibodies, we could only detect diffuse cytosolic EspM2 staining (data not shown) while, using vinculin antibodies, we observed intense distal staining of the GP-SF (Fig. 2), suggesting that the GP-SF triggered by EspM2 are linked to the plasma membrane through focal adhesions (Ziegler *et al.*, 2006).

Infection of Swiss 3T3 cells with E2348/69 expressing EspM1 (B171) (Fig. 2), EspM1 (Sakai) and TrcA (data not shown) resulted in simultaneous formation of actin pedestals and localized parallel stress fibres (LP-SF), which were subtly different from those triggered by EspM2 as they were confined to the site of bacterial adhesion (Fig. 2). Inmunofluorescence using the anti-vinculin antibodies revealed intense staining at the tip of each of the LP-SF (Fig. 2). Swiss cells infected with wild-type B171 for 90 min exhibited widespread stress fibres of the LP-SF morphology alongside well-developed actin pedestals (data not shown).

Infection of Swiss 3T3 with E2348/69 expressing EspM3 (CR) resulted in simultaneous formation of actin pedestals and stress fibres with vinculin-rich tips in *c*. 90% of the cells at 1.5 h post infection (Fig. 2). EspM3 triggered formation of a distinct architecture of localized radial (3D) stress fibres (LR-SF) (Fig. 2); despite the localized nature of the stress fibres, using anti-HA antibodies we only observed a diffuse cytosolic EspM3 straining (data not shown). To our knowledge, LR-SF have not been previously described in an infection context, but are reminiscent of cells treated with phorbal esters such as TPA (Imamura *et al.*, 1998).

The GP-SF, LP-SF and LR-SF appeared as soon as 30 min post infection and were stable for at least 3 h (data not shown). In control experiments, Swiss 3T3 cells were infected with E2348/69Δ*escN* (T3SS mutant), E2348/69Δ*map*, E2348/69Δ*tir* and E2348/69Δ*espG*/*espG2* expressing EspM2 (Sakai). While no GP-SF or actin pedestal structures were seen after infection with the E2348/69Δ*escN* mutant, GP-SF and actin pedestals, similar to those seen after infection with wild-type E2348/69, were seen after infection with all other mutant strains. In addition, ectopic expression of EspM2 in transfected cells also triggers formation of GP-SF (data not shown).

Taken together, these results illustrate that the A/E EspM WxxxE effectors subvert actin dynamics and trigger formation of GP-SF (EspM2), LP-SF (EspM1 and TrcA) or LR-SF (EspM3). We selected EspM2 (Sakai) and EspM3 as model effectors for further study.

### The effect of site-directed mutagenesis on the activity of EspM2 and EspM3

**Fig. 3:**
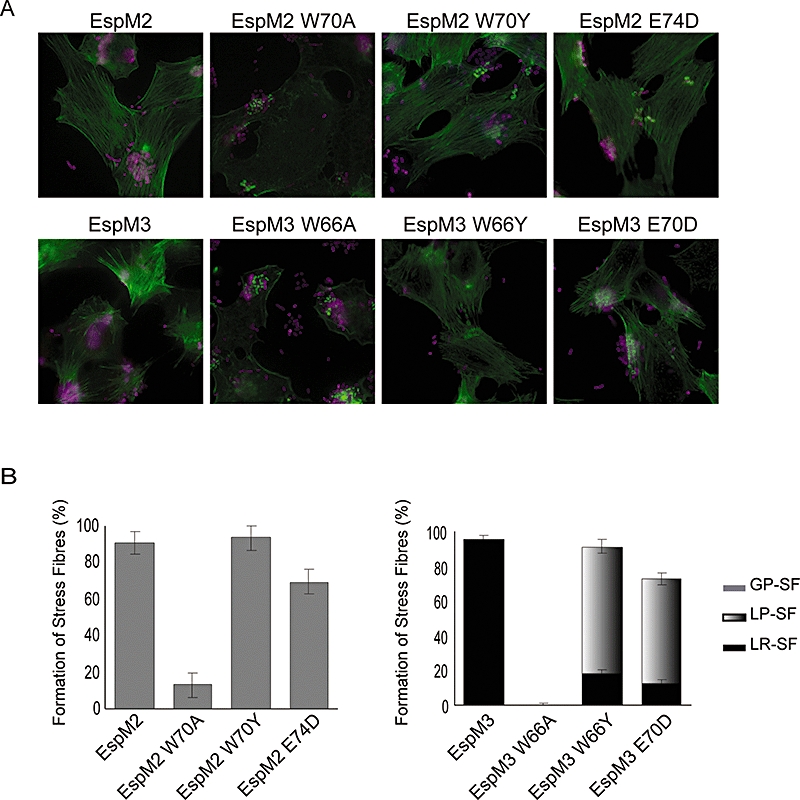
Effect of site-directed mutagenesis on stress-fibre formation. A. Merged fluorescence microscopy of Swiss 3T3 cells infected for 90 min with primed E2348/69 expressing EspM2 (Sakai) and EspM3 (CR) derivative mutants within the conserved WxxxE motif. Actin was stained with Oregon green phalloidin and EPEC were detected with rabbit anti-0127 antibody. B. Quantification of stress-fibre formation in Swiss cells after 90 min infection. One hundred infected cells were counted in triplicate from three independent experiments. Results are presented as mean ± SEM.

The actin modulation activity of the WxxxE effectors Map, IpgB1 and IpgB2 were previously shown to be dependent on their invariant WxxxE motif (Alto *et al.*, 2006). To determine if this is also true for EspM2 and EspM3, the conserved residues W70 and W66, respectively, were substituted by Ala. Swiss cells infected with E2348/69 expressing EspM2_W70A_ and EspM3_W66A_ triggered actin-rich pedestals with no evidence of GP-SF or LP-SF respectively, and were indistinguishable from cell infected with wild-type E2348/69 (Fig. 3). Conversely, conservative substitution of the Trp and Glu residues with a Tyr and Asp, respectively, did not result in any significant loss of stress-fibre formation (Fig. 3). These substitutions did, however, produce a shift in the architecture of stress fibres induced by EspM3 from a LR-SF morphology to a LP-SF arrangement. In addition, we mutated additional conserved residues located within the core region of the WxxxE effectors (Fig. 1). Mutagenesis of L114, D115 and H125 of EspM3 to Ala also resulted in a similar conformational change in the stress fibres (data not shown). Mutagenesis of other conserved residues within this group of effectors in EspM3 (Fig. 1) did not have any significant effect on stress-fibre formation or morphology (data not shown).

### EspM2 and EspM3 activate RhoA

**Fig. 4:**
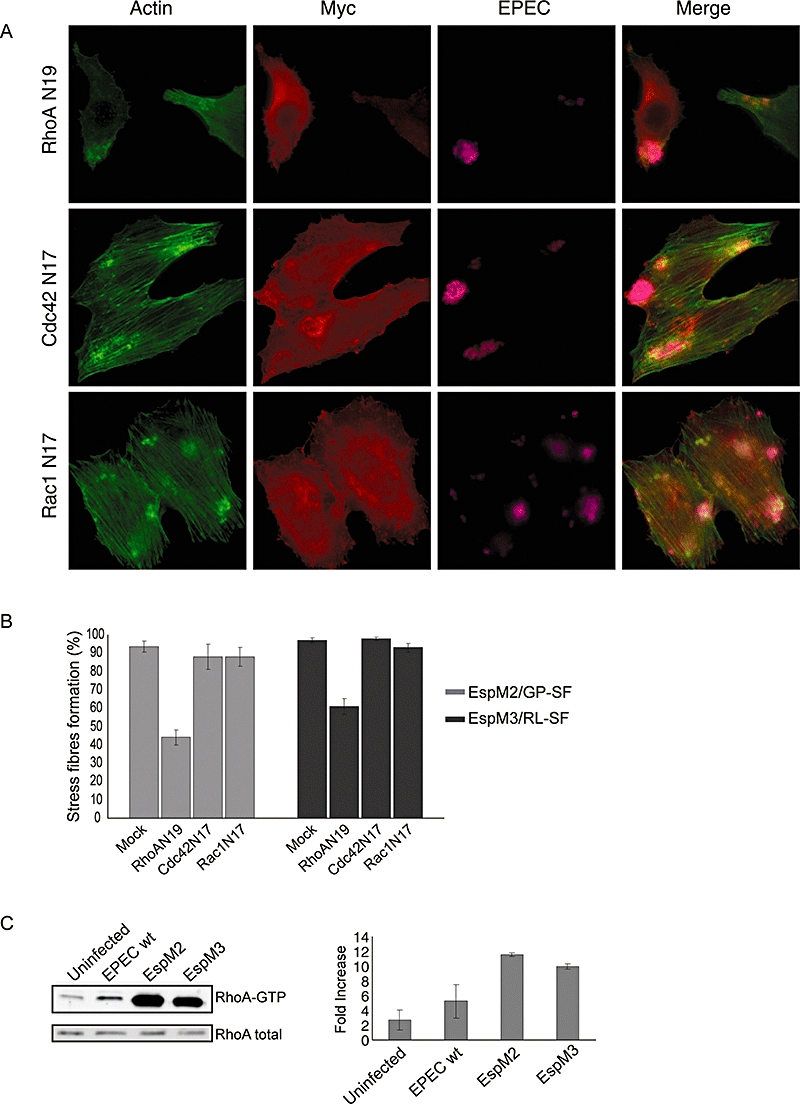
Stress-fibre formation by EspM2 and EspM3 is RhoA-dependent. A. Swiss 3T3 cells were transfected with dominant negative GTPase contracts 24 h prior to infection with E2348/69 expressing EspM2. Actin was stained with Oregon green phalloidin, the Myc-tagged GTPases with mouse anti-myc and EPEC with rabbit anti-O127. Actin-rich pedestals are observed under adherent bacteria in all transfected cells. GP-SF are observed, at the level of mock transfection, in cells transfected with dominant negatives Cdc42^N17^ or Rac1^N17^. In contrast, transfection with Rho^N19^ inhibited formation of GP-SF. B. Quantification of stress-fibre formation in transfected Swiss 3T3 cells infected for 90 min with E2348/69 expressing EspM2 or EspM3. Formation of stress fibres in one hundred transfected infected cells were counted in triplicate from three independent experiments. Results are presented as mean ± SEM. Formation of stress fibres depending on EspM2 or EspM3 is affected by expression of RhoA^N19^, but not by expression of Cdc42^N17^ or Rac^N17^. C. Swiss 3T3 cells were infected with wild-type E2348/69 or E2348/69 expressing EspM2 or EspM3. Cells were lysed and a GST fusion of the Rho binding domain of Rhotekin was used to co-purify RhoA-GTP. Total RhoA in the lysates and RhoA-GTP were detected by Western blot with anti-RhoA antibodies. Quantified data are means ± SD of the results of three independent experiments.

The formation of actin stress fibres in eukaryotic cells is regulated by the GTP-binding protein RhoA (Jaffe and Hall, 2005). However, IpgB2 has been shown to trigger formation of stress fibres through a mechanism which is independent of RhoA (Alto *et al.*, 2006). In order to determine whether formation of GP-SF or LR-SF by EspM2 and EspM3 is dependent on activation of small Rho GTPases, we transfected Swiss cells with dominant negative Cdc42^N17^, Rac1^N17^ and RhoA^N19^ (which competitively and specifically inhibit activation of the small GTPases). Infection of transfected cells with E2348/69 expressing EspM2 or EspM3 for 1.5 h revealed that formation of GP-SF and LR-SF, respectively, was not affected by inactivation of Cdc42 or Rac1 (Fig. 4A and B). In contrast, infection of cells expressing RhoA^N19^ resulted in formation of typical actin-rich pedestals, but with a marked reduction in formation of GP-SF or LR-SF respectively (Fig. 4A and B). This suggests that, unlike IpgB2, formation of stress fibres by EspM2 and EspM3 is RhoA-dependent.

In order to determine if EspM2 and EspM3 activate RhoA, we performed pull-down assays using GST-Rhotekin, which specifically binds the active GTP-bound form of RhoA. Cells incubated with 20 μM nocodazole, which destabilizes microtubules (DeBrabander *et al.*, 1976), for 40 min (data not shown), were used as a positive control and cell infected with wild-type E2348/69 were used as negative control. Swiss 3T3 cells infected with E2348/69 expressing EspM2 or EspM3 exhibited a significantly higher level of activated RhoA compared with cells infected with the wild-type E2348/69 (Fig. 4C). These results suggest that formation of GP-SF and LR-SF is dependent on RhoA signalling.

### EspM2 and EspM3 stimulate the Rho-ROCK pathway

**Fig. 5:**
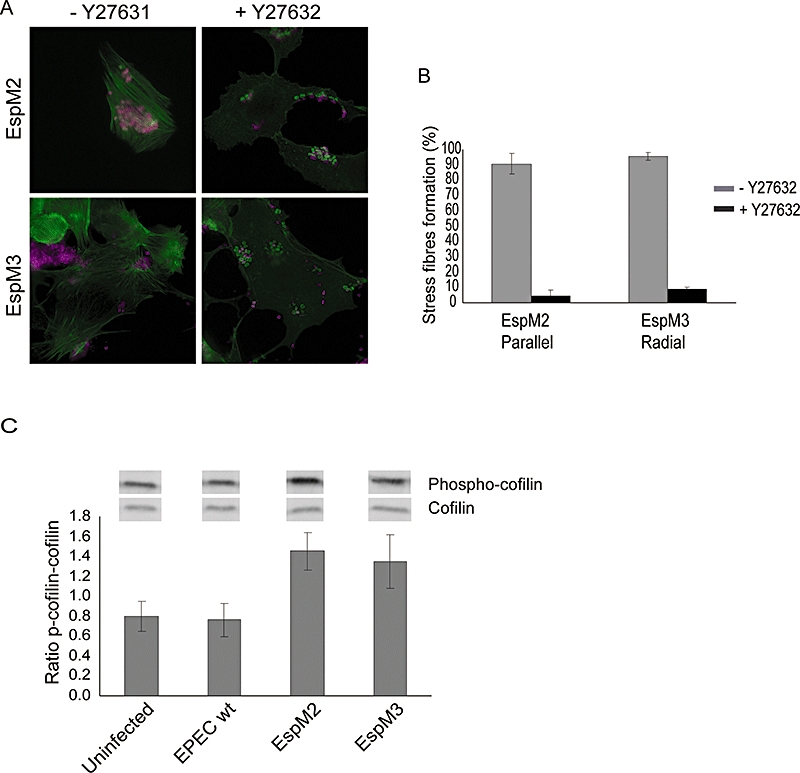
EspM2 and EspM3 trigger the ROCK-LIMK-cofilin pathway. A. Fluorescence microscopy of Swiss 3T3 cells incubated for 1 h in presence or absence of 10 μM of the ROCK inhibitor Y-27632 and infected for 90 min with E2348/69 expressing EspM2 or EspM3. Y-27632 did not affect formation of actin-rich pedestals, but abolished formation of stress fibres. B. Percentage of Swiss 3T3 cells presenting stress fibres in the presence or absence of Y-27632. One hundred cells were counted in triplicate from three independent experiments. Results are presented as mean ± SEM. C. Swiss 3T3 cells were infected for 90 min with wild-type E2348/69 or E2348/69 expressing EspM2 or EspM3. The levels of cofilin and phospho-cofilin were determined by Western blot using anti-cofilin and anti-phospho-cofilin antibodies. Quantification of densitometry was conducted using image J software and results presented here represent the mean ratio ± SD of four independent experiments.

The p160 Rho-associated coiled-coil-containing protein kinase (ROCK) is one of the main RhoA downstream effectors which mediates the formation of stress fibres (Jaffe and Hall, 2005). To determine if EspM2 and EspM3 activate the RhoA-ROCK pathway, Swiss 3T3 cells were incubated with the ROCK inhibitor Y-27632 for 1 h prior to infection with E2348/69 expressing EspM2 or EspM3. Y-27632 is a highly specific inhibitor of ROCK-I and ROCK-II which competitively excludes ATP from the catalytic site (Yamaguchi *et al.*, 2006). Pre-incubation with Y-27632 completely blocked formation of GP-SF and LR-SF by EspM2 and EspM3 respectively (Fig. 5). The ROCK inhibitor did not affect formation of actin-rich pedestals.

LIM kinase (LIMK) is downstream effector of ROCK which is involved in stress-fibres formation (Jaffe and Hall, 2005). It has been shown that phosphorylation of the LIMK by ROCK and the consequent inactivation (by phosphorylation) of cofilin by LIMK contribute to Rho-induced formation of stress fibres (Maekawa *et al.*, 1999). We examined the phosphorylation state of cofilin by immunoblot following infection of Swiss cell with E2348/69 expressing EspM2 or EspM3. When compared with uninfected or cells infected with the wild-type E2348/69, higher level of phospho-cofilin was observed after translocation of EspM2 or EspM3 (Fig. 5). These results suggest that EspM2 and EspM3 trigger stress-fibre formation through the RhoA-ROCK-LIMK-cofilin pathway.

## Discussion

In 2006, Alto *et al.* (2006) assembled several known T3SS effectors into a single family that share the common motif Trp-xxx-Glu (WxxxE). They suggested that IpgB1, IpgB2 and Map (the founder WxxxE effectors) mimic, in a GTP-independent mechanism, the activated form of Rac-1, RhoA and Cdc42 respectively. In this study, we report the discovery and characterization of an array of T3SS effector proteins in A/E pathogens which belong to the WxxxE family. These new effectors share a high degree of homology with each other (47–86% identity) and with the *Shigella* effector IpgB2 (40–47% identity).

The founder WxxxE effectors are potent modulators of the host actin cytoskeleton (Kenny *et al.*, 2002; Alto *et al.*, 2006; Handa *et al.*, 2007). In order to determine if the new proteins we studied are true WxxxE effectors that can subvert actin dynamics, we utilized EPEC EPEC234/69 as a vehicle for protein translocation into Swiss cells. EPEC234/69 is a suitable host as it only harbours the WxxxE effector Map, which induces transient filopdia at early time points post infection (Kenny and Jepson, 2000). Using this infection model, we demonstrated that overexpression of EspM1, EspM2, EspM3 and TrcA in EPEC E2348/69 triggers formation of stress fibres, related to those induced by the *Shigella* effector IpgB2 (Alto *et al.*, 2006). Importantly, the stress fibres triggered by these WxxxE effectors had distinct architectures. Although the EspM homologues were expressed from isogenic plasmids, we cannot exclude the possibility that differences in expression levels or translocation efficiency might contribute to their distinct stress fibre morphologies which appear as GP-SF throughout the cell cytosol for EspM, and LP-SF restricted to the site of bacterial attachment in the case of EspM1 and TrcA. Most striking was the phenotype associated with translocation of EspM3, which induced formation of LR-SF, which are reminiscent of the phenotypes linked to constitutively active Rho Kinase and phobal ester treatment such as TPA (Imamura *et al.*, 1998). To our knowledge, this is the first time that such a phenotype has been described in an infection context. Despite the global and localized stress fibres triggered by EspM2 and EspM1 and EspM3, respectively, all proteins were found diffusely in the cytsol. Vinculin was found at the tip of the WxxxE effector-triggered stress fibres, suggesting that they are linked to the plasma membrane via focal adhesion. The molecular basis for the different stress fibre architectures triggered by EspM1, EspM2, EspM3 and TrcA is not currently known. Of note is the fact that TrcA (bfpT-regulated chaperone-like protein gene) was originally reported to bind the pilin protein BfpA and the outer membrane adhesion intimin (Tobe *et al.*, 1999), suggesting that the TrcA might have a role in the bacterial cytosol prior to translocation into the host cell.

As EspM2 and EspM3 trigger the two extreme stress fibre phenotypes, they were chosen for detailed functional analysis. We confirmed that the GP-SF and LR-SF triggered by EspM2 and EspM3 are dependent on the conserved WxxxE motif via substitution of the conserved Trp with an Ala. However, using conservative substitutions, Tyr and Asp for the Trp and Glu, respectively, we did not observe any significant loss in biological activity. Translocation of EspM2 YxxxE and EspM2 WxxxD resulted in global stress-fibre formation, while translocation of EspM3 YxxxE and WxxxD resulted in formation of mainly LP-SF as compared with the LR-SF observed in the wild-type phenotype. The reason for the change of morphology observed for EspM3 YxxxE and WxxxD is currently not known.

These results suggest that the biological activity of the WxxxE proteins is not reliant upon the presence of the conserved tryptophan or glutamic acid, but rather the presence of a bulky aromatic residue and an amino acid carrying a carbocylic acid side group. Carboxylic side groups are involved in a range of biological functions such as protease activity (Kakudo *et al.*, 1992) and interactions particularly with positively charged moieties such as metal ions.

Further alanine substitutions of conserved residues did not abolish stress-fibre formation, suggesting that none of the selected residues on their own, other than the Typ and Glu, are essential for function. Structural data will be essential in order to gain further insights into the mechanism by which the conserved motif and surrounding residues lead to formation of the plethora actin structures associated with this family of effector proteins.

Using specific E2348/69 mutants, we have shown that stress-fibre formation was dependent on type III secretion but independent of other T3SS effectors, including Map, Tir, and EspG and EspG2. The later two were shown to induce late RhoA-dependent stress fibres by liberation of an active form of the RhoA-specific GEF, GEF-H1, as a result of disruption of the host cell microtubule network (Matsuzawa *et al.*, 2004). This EspG activity might represent the small percentage (15%) of stress fibre seen in Swiss cells infected with wild-type E2348/69. The fact that EspM2 is necessary and sufficient for stress-fibre formation is supported by the fact that transfection of EspM2 into HeLa cells resulted in formation of characteristic GP-SF (data not shown).

The Ras superfamily of the small GTPases is the target of many bacterial effectors and toxins because of its role in the control of a wide range of cellular processes including cytoskeletal dynamics, membrane trafficking and growth. Several T3SS effectors, such as YopT from *Yersinia* spp., are known to directly modify the small GTPases (Wong and Isberg, 2005; Fueller *et al.*, 2006). Other effectors can either modify or mimic GEFs or GAPs (Finlay, 2005). Although sharing no sequence homology with the Rho GTPases, Alto *et al.* (2006) suggested that IpgB1, IpgB2 and Map mimic, in a GTP-independent mechanism, the activated form of Rac1, RhoA and Cdc42, respectively. They showed that stress-fibre formation was RhoA-independent as IpgB2 was functional in the presence of RhoA inhibitors (e.g. C3 botulinum toxin) or dominant negative RhoA^T19N^. Moreover, it was shown that IpgB2 interacts with two of the major effectors of RhoA, namely mDIA and ROCK, activating the latter directly.

Alto *et al.* (2006) reported that transfection of IpgB1 stimulated formation of actin-rich membrane ruffles at the dorsal cell surface, which resembled membrane ruffles induced by Rac-1. Dorsal ruffles were not produced by IpgB1^E80A^. A recent study by Handa *et al.* (2007) has shown that IpgB1 mimics RhoG and triggers membrane ruffling by binding ELMO and recruiting the ELMO–Dock180 complex to the membrane where it functions as a GEF for Rac1.

In this investigation, we found that EspM2 and EspM3 activate RhoA. Using dominant negative RhoA^T19N^, we observed a 60% and 40% reduction in stress-fibre formation after infection of Swiss cell with E2348/69 expressing EspM2 and EspM3 respectively. In agreement with Alto *et al.* (2006), using the specific ROCK inhibitor Y27632, we showed that the stress fibres induced by EspM2 and EspM3 are totally dependent upon ROCK activity. Consistent with this, we have shown that EspM2 and EspM3 trigger phosphorylation of cofilin, a classical downstream ROCK target. Although our data do not exclude the possibility that EspM2 and EspM3 are capable of mimicking RhoA to some degree, these effectors appear to contribute to stress-fibre production by activating endogenous RhoA. Alternatively, as was recently shown for IpgB1 (Handa *et al.*, 2007), EspM might mimic GTPases upstream of RhoA activation. Further studies are needed to determine the mechanism of RhoA activation by EspM.

## Experimental procedures

### Bacterial strains, growth conditions and cell culture

The bacterial strains used in this study are listed in Table 1. Bacteria were grown from single colonies in Luria–Bertani (LB) broth in a shaking incubator at 37°C or maintained on LB plates. Culture media was supplemented with Ampicillin (100 μg ml^−1^) as appropriate.

**Table 1:** List of strains.

Strain	Description	Source/reference
E2348/69	Wild-type EPEC O127:H6	Levine *et al.* (1978)
ICC168	*C. rodentium*	Barthold *et al.* (1976); Wiles *et al.* (2004)
EHEC Sakai	Wild-type EHEC O157:H7 (RIMD 0509952)	Hayashi *et al.* (2001)
EPEC B171	Wild-type EPEC O111:NM	Riley *et al.* (1987)
ICC192	EPEC E2348/69Δ*escN*	Garmendia *et al.* (2004)
ICC202	EPEC E2348/69Δ*map*	Simpson *et al.* (2006)
ICC225	EPEC E2348/69Δ*tir*	Bai *et al.* (2008)
ICC243	EPEC E2348/69Δ*espG-espG2*	Marchès *et al.* (2008)

Bacterial cultures were primed prior to infection by growth in Dulbecco's modified Eagles media (DMEM) with 4500 mg ml^−1^ glucose supplemented with 1% mannose for 3 h (Collington *et al.*, 1998) before addition of 1 mM IPTG to induce protein expression.

Swiss NIH 3T3 cells were maintained in DMEM with 4500 mg ml^−1^ glucose and supplemented with 10% fetal calf serum (Gibco) and 4 mM Glutamax (Gibco).

### Bioinformatics

A psi-blast search (Altschul *et al.*, 1997) was performed under default conditions using IpgB2 from *Shigella flexneri* (gi:13448971) and Map from EPEC (gi:2865296) as query sequence to search the latest version of the NCBI NR database and combined with a library of peptide sequences derived from all coding sequences ≥ 50 codons in length from the genome sequences of *C. rodentium* ICC168, EPEC B171 and EPEC E2348/69.

### Multiple alignment and phylogenetic analysis of WxxxE effectors

Using the sequence alignment program Multalign, a radial phylogenetic tree was constructed based upon hierarchical clustering of the global sequence homology of the A/E pathogen group WxxxE proteins and IpgB1 compared with that of IpgB2. Protein sequences identified in the blast search that did not show near full-length homology to other EspM proteins were excluded from further analysis on the grounds that they are likely to represent non-functional pseudogenes.

### Plasmids and molecular techniques

Plasmids used in this study are listed in Table 2; primers are listed in Table 3. The genes encoding effector proteins were amplified by PCR from genomic DNA and cloned into pSA10 with a C-terminal HA tag. *espM2* and *espM1* genes were amplified from EHEC O157:H7 strain Sakai, *trcA* and *espM1* genes were amplified from EPEC O111:NM strain B171 and *espM2* and *espM3* genes were amplified from *C. rodentium*. All constructs were verified by DNA sequencing.

**Table 2:** List of plasmids.

Name	Description	Reference
pSA10	pKK177-3 with Lac^q^	Schlosser-Silverman *et al.* (2000)
pICC398	pSA10::*espM2* from O157:H7 Sakai fused to HA	This study
pICC399	pSA10::*espM3* from *C. rodentium* ICC169 fused to HA	This study
pICC400	pSA10::*espM2* from *C. rodentium* ICC169 fused to HA	This study
pICC401	pSA10::*espM1* from O157:H7 Sakai fused to HA	This study
pICC402	pSA10::*trcA* from O111:NM B171 fused to HA	This study
pICC403	pSA10::*espM1* from O111:NM B171 fused to HA	This study
pICC404	pSA10::*espM2-*W70A fused to HA	This study
pICC405	pSA10::*espM2-*W70Y fused to HA	This study
pICC406	pSA10::*espM2-*E74D fused to HA	This study
pICC407	pSA10::*espM3-*W66A fused to HA	This study
pICC408	pSA10::*espM3-*W66D fused to HA	This study
pICC409	pSA10::*espM3*-S9A + S12A fused to HA	This study
pICC410	pSA10::*espM3-*L114A fused to HA	This study
pICC411	pSA10::*espM3-*D115A fused to HA	This study
pICC412	pSA10::*espM3-*H125A fused to HA	This study
pICC413	PSA10::*espM3-*K149A fused to HA	This study
	pDEST53::*espM2*	Invitrogen, This study
	pRK5::*myc-rhoA*^*N17*^	Pelletier *et al.* (2005)
	pRK5::*myc-rac1*^*N17*^	Pelletier *et al.* (2005)
	pRK5::*myc-cdc42*^*N17*^	Pelletier *et al.* (2005)
	pGex::*gst-RBD*	Sander *et al.* (1999)

**Table 3:** List of primers.

Primer	Sequence
EspM3-S9A_S12A-F	5′-ccagtgaatgttcaaggtttagcttttaatgcttttggtatcagctgccatc-3′
EspM3-S9A_S12A-R	5′-gatggcagctgataccaaaagcattaaaagctaaaccttgaacattcactgg-3′
EspM3-W66A-F	5′-gcagagtactaaagatattgatggagcgataaaagatgaacagaaagtgtat-3′
EspM3-W66A-R	5′-atacactttctgttcatcttttatcgctccatcaatatctttagtactctgc-3′
EspM3-H125A-F	5′-ggagcagctcaaagctccatcaatgctatgataataggaaatggtt-3′
EspM3-H125A-R	5′-aaccatttcctattatcatagcattgatggagctttgagctgctcc-3′
EspM3-Y188F-F	5′-gtatgattcctcgttatatgaggtttgctatatcgctgtacc-3′
EspM3-Y188F-R	5′-ggtacagcgatatagcaaacctcatataacgaggaatcatac-3′
EspM3-D115A-F	5′-ttatggcctggaacttgcttctggagcagctcaaa-3′
EspM3-D115A-R	5′-tttgagctgctccagaagcaagttccaggccataa-3′
EspM3-L114A-F	5′-tttcaaagcattatggcctggaagctgattctggagcagc-3′
EspM3-L114A-R	5′-gctgctccagaatcagcttccaggccataatgctttgaaa-3′
EspM2Cr-W66A-F	5′-ggcagagcactaaagatcttttagcagcgaaaaaagatgaaaaagtaatttatc-3′
EspM2Cr-W66A-R	5′-gataaattactttttcatcttttttcgctgctaaaagatctttagtgctctgcc-3′
EspM2Cr-H125A-F	5′-gcccgctcagcacaaagttctataaatgctatcataaccggaaa-3′
EspM2Cr-H125A-R	5′-tttccggttatgatagcatttatagaactttgtgctgagcgggc-3′
EspM3-K149A-F	5′-cgaggggatgaccaggaatgtagcaaatcatacatcagattacatg-3′
EspM3-K149A-R	5′-catgtaatctgatgtatgatttgctacattcctggtcatcccctcg-3′
EspM3-W66Y-F	5′-gcagagtactaaagatattgatggatatataaaagatgaacagaaagtgtatcc-3′
EspM3-W66Y-R	5′-ggatacactttctgttcatcttttatatatccatcaatatctttagtactctgc-3′
EspM3-E70D-F	5′-tgatggatggataaaagatgatcagaaagtgtatccatcaagg-3′
EspM3-E70D-R	5′-ccttgatggatacactttctgatcatcttttatccatccatca-3′
EspM2-W70Y-F	5′-gcagagtactaaagatattgatgagtatataaaagatgaacggatagtatatcc-3′
EspM2-W70Y-R	5′-ggatatactatccgttcatcttttatatactcatcaatatctttagtactctgc-3′
EspM2-E74D-F	5′-taaagatattgatgagtggataaaagatgatcggatagtatatccctc-3′
EspM2-E74D-R	5′-gagggatatactatccgatcatcttttatccactcatcaatatcttta-3′
EspM1-pKK-F	5′-ttgaattcatgccagtaaatgcgacagg-3′
EspM1-pKK-R	5′-ccaatgcactgcagttaagcgtagtctgggacgtcgtatgggtacccctgtataacacgactca-3′
EspM2-pKK-F	5′-tttgaattcatgccgatgaatactacaggtatgt-3′
EspM2-pKK-R	5′-tttctgcagtcaagcgtagtctgggacgtcgtatgggtatccctgtatagcacgcatcaa-3′
EspM2 W70A-F	5′-gcagagtactaaagatattgatgaggcgataaaagatgaacggatagtatat-3′
EspM2 W70A-R	5′-atatactatccgttcatcttttatcgcctcatcaatatctttagtactctgc-3′
EspM3-pKK-F	5′-ttgaattcatgccagtgaatgttcaagg-3′
EspM3-pKK-R	5′-ttgcctgcagttaagcgtagtctgggacgtcgtatgggtacacgatatagcatacctca-3′
EspM2Cr-pKK-F	5′-ttgaattcatgccagcgaatatatcagg-3′
EspM2Cr-pKK-R	5′-ttgtcgacttaacttaagcgtagtctgggacgtcgtatgggtatgccgacatggcatttgcca-3′
TrcA-pKK-F	5′-ttgaattcatgccgatgaatactacaagtac-3′
TrcA-pKK-R	5′-aactgcagttaagcgtagtctgggacgtcgtatgggtatgcctgtatagcacacctca-3′
EspM1 B171-pKK-F	5′-tacccatacgacgtcccagactacgct-3′
EspM1 B171-pKK2-R	5′-ccaatgcactgcagttaagcgtagtctgggacgtcgtatgccgatatgacgctggcag-3′

The mammalian expression vector pRK5 containing one of Rho^N19^, Rac^N17^ or Cdc42^N17^ dominant negatives used in the transfection assays was a gift from Nathalie Lamarche-Vane.The vector pGEX expressing the binding domain of Rhotekin fused to GST was a gift from J. Bertoglio.

### Site-directed mutagenesis

Site-directed mutagenesis was carried out using a Quickchange II kit (Stratagene) according to the manufacturer's instructions. Primers were designed using the Quickchange mutagenic primer design program (Stratagene). Plasmids pSA10 containing *espM1*, *espM2*, *espM3 and trcA* were used as templates for the mutagenic reactions. Colonies were screened by sequencing and alignment to wild-type sequences to confirm mutagenesis.

### Infection of Swiss 3T3 cells with EPEC E2348/69

Forty-eight hours prior to infection, Swiss 3T3 cells were seeded onto glass coverslips at a density of approximately 5 × 10^5^ cells per well and maintained in DMEM 4500 supplemented with 10% FCS at 37°C in 5% CO_2_. Three hours before infection, the cells were washed three times with PBS and the media replaced with fresh DMEM 4500 without FCS supplemented with 1% mannose. Overnight cultures of the appropriate bacteria were inoculated 1:50 into DMEM and primed as described previously (Collington *et al.*, 1998). Five hundred microlitres of primed bacteria was added to the wells and infections were carried out at 37°C in 5% CO_2_ for 1.5 h.

### Transfection

Swiss 3T3 cells were transfected with the mammalian expression vector pRK5 containing one of RhoA^N19^, Rac^N17^ or Cdc42^N17^ dominant negatives fused to a Myc tag by lipofectamine2000 (Invitrogen) according to the manufacturer's recommendations. The cells were incubated at 37°C in a humidified incubator with 5% CO_2_ for 24 h, washed twice in PBS before having their media replaced with DMEM as described previously. Transfected cells were infected with the appropriate strain as described above.

### Immunofluorescence staining and microscopy

Coverslips were washed three times in PBS and fixed with 3% Paraformaldehyde for 15 min before washing three more times in PBS. For immunostaining, the cells were permeabilized for 5 min in PBS 0.5%/Triton X-100, washed three times in PBS and quenched for 30 min with 50 mM NH_4_Cl. The coverslips were then blocked for 1 h with PBS/0.5% BSA before incubation with primary and secondary antibodies. The primary antibody Mouse αVinculin (Abcam) and mouse anti-Myc (Millipore) were used at a dilution of 1:200, while Rabbit αO127 was used at a dilution of 1:150. Coverslips were incubated with the primary antibody for 1 h, washed three times in PBS and incubated with the secondary antibodies. Donkey anti-rabbit IgG conjugated to a Cy5 fluorophore or Donkey anti-mouse IgG conjugated to a Cy3 fluorophore (Jackson laboratories) was used at a 1:200 dilution. Actin was stained using Oregon Green phalloidin (Invitrogen) at a 1:100 dilution. All dilutions were in PBS/0.5% BSA. Coverslips were mounted on slides using ProLong Gold anti-fade reagent (Invitrogen) and visualized by Zeiss Axioimager immunofluorescence microscope (100× objective giving a total magnification of 1000×) using the following excitation wavelengths: Cy3 – 605 nm, Cy5 – 690 nm and Oregon Green – 525 nm. All images were analysed using the Axiovision Rel 4.5 software and trimmed to 5 cm^2^ (300 pixels) using Adobe photoshop.

### Chemical inhibition of ROCK activity

Coverslips were set up for infection as described above. One hour prior to infection, Y-27632 (Sigma) was added to cells to attain a final concentration of 10 μM per well. Coverslips were infected and processed for immunofluorescence.

### Quantification of cofilin phosphorylation

Forty-eight hours prior to infection, Swiss 3T3 cells were seeded onto 6-well plates to attain confluent monolayer. Each well was infected as described above. After a 1.5 h infection, cells were lysed by addition of 4× protein SDS-PAGE loading buffer. The lysate was harvested and heated at 100°C for 5 min before loading in duplicate onto a 12% SDS-PAGE gel. The proteins were then transferred to PVDF by wet transfer. The PVDF membranes were blocked overnight in TBS 5%/BSA at 4°C with gentle rocking. Each membrane was incubated in either monoclonal mouse anti-cofilin (Cell Signalling Technology) or mouse anti-Phospho-cofilin (Cell Signalling Technology) at a dilution of 1:1000 in TBS 5%/BSA for 1 h at room temperature with gentle rocking. The membranes were washed five times in TBS 1%/Tween for 5 min and incubated for 45 min with a 1:3000 dilution of Goat α Mouse (Invitrogen) secondary antibody coupled to HRP at room temperature. The membranes were developed using ECL reagents (GE healthcare) before detection using chemiluminescence in a LAS 3000 Fugi imager. Western blots were analysed by densitometry using ImageJ software. Results presented are the average of five independent experiments.

### Preparation of GST-Rhotekin and RhoA-GTP pull-downs

An overnight culture of *E. coli* BL21 expressing pGEX encoding the RhoA binding domain of Rhotekin was diluted 1:20 and cultured at 30°C until OD_260nm_ reached 0.7; the culture was induced with 1 mM IPTG and incubated for a further 4 h. The bacterial culture was aliquoted into 50 ml falcon tubes and centrifuged for 15 min at 4600 r.p.m. at 4°C and the pellets stored at −80°C. The pellets were resuspended in lysis buffer [20% Saccharose, 10% glycerol, 50 mM Tris pH 8, 200 mM Na_2_S_2_O_3,_ 2 mM MgCl_2_, 2 mM DTT and 1% protease inhibitor cocktail (Sigma)] and sonicated five times for 10 s. The solution was centrifuged for 30 min at 15 000 r.p.m. at 4°C and the supernatants harvested and coupled to GST-glutathione S transferase beads for 45 min at 4°C.

The 75 cm^2^ cell culture flasks were seeded with Swiss 3T3 cells, infected as described above and the cells lysed in 750 μl of Mg^++^ buffer [25 mM Hepes, pH 7.5, 150 mM NaCl, 1% NP-40, 10 mM MgCl_2_, 5% glycerol, 1 mM EDTA and protease inhibitors (Sigma)]. The lysate was transferred to a pre-chilled eppendorf tube and centrifuged at 14 000 r.p.m. for 5 min at 4°C. The cleared lysate was transferred to a fresh pre-chilled eppendorf tube containing 30 μl of GST-Rhotekin-RBD beads. The lysate was incubated with the beads for 1 h at 4°C. The suspension was washed three times in Mg^++^ buffer spinning down at 14 000 r.p.m. at 4°C between each wash. The beads were eluted by the addition of 45 μl of 2× protein loading buffer and the samples heated at 100°C for 5 min. The samples were loaded on a 15% SDS-PAGE gel. The gels were transferred to PVDF and blotted as previously described using monoclonal RhoA antibody (Santa Cruz).

## References

[b1] Alto NM, Shao F, Lazar CS, Brost RL, Chua G, Mattoo S (2006). Identification of a bacterial type III effector family with G protein mimicry functions. Cell.

[b2] Altschul SF, Madden TL, Schäffer AA, Zhang J, Zhang Z, Miller W, Lipman DJ (1997). Gapped BLAST and PSI-BLAST: a new generation of protein database search programs. Nucleic Acids Res.

[b3] Bai L, Schüller S, Whale A, Mousnier A, Marches O, Wang L (2008). Enteropathogenic *Escherichia coli* (EPEC) O125: H6 triggers attaching and effacing lesions on human intestinal biopsies independently of Nck and TccP/TccP2. Infect Immun.

[b4] Barthold SW, Coleman GL, Bhatt PN, Osbaldiston GW, Jonas AM (1976). The etiology of transmissible murine colonic hyperplasia. Lab Anim Sci.

[b5] Beuzon CR, Meresse S, Unsworth KE, Ruiz-Albert J, Garvis S, Waterman SR (2000). *Salmonella* maintains the integrity of its intracellular vacuole through the action of SifA. EMBO J.

[b6] Campellone KG, Robbins D, Leong JM (2004). EspF_U_ Is a translocated EHEC effector that interacts with Tir and N-WASP and promotes Nck-independent actin assembly. Dev Cell.

[b7] Charpentier X, Oswald E (2004). Analysis of type III translocation signals of enteropathogenic and enterohemorrhagic *Escherichia coli* effectors using TEM-1 beta-lactamase as a fluorescence-based reporter. J Bacteriol.

[b8] Collington GK, Booth IW, Knutton S (1998). Rapid modulation of electrolyte transport in Caco-2 cell monolayers by enteropathogenic *Escherichia coli* (EPEC) infection. Gut.

[b9] Corpet F (1988). Multiple sequence alignment with hierarchical clustering. Nucleic Acids Res.

[b10] Cossart P, Sansonetti PJ (2004). Bacterial invasion: the paradigms of enteroinvasive pathogens. Science.

[b11] DeBrabander MJ, Van de Viere FEM, Aerts M, Borgers PA, Jannsen PA (1976). The effects of nocodazole, a new synthetic antitumoral drug interfering with microtubules, on mammalian cells cultured in vitro. Cancer Res.

[b12] Finlay BB (2005). Bacterial virulence strategies that utilize Rho GTPases. Curr Top Microbiol Immunol.

[b13] Fueller F, Bergo MO, Young SG, Aktories K, Schmidt G (2006). Endoproteolytic processing of RhoA by Rce1 is required for the cleavage of RhoA by *Yersinia enterocolitica* outer protein T. Infect Immun.

[b14] Galan JE, Wolf-Watz H (2006). Protein delivery into eukaryotic cells by type III secretion machines. Nature.

[b15] Garmendia J, Phillips A, Chong Y, Schuller S, Marches O, Dahan S (2004). TccP is an enterohaemorrhagic *E. coli* O157:H7 type III effector protein that couples Tir to the actin-cytoskeleton. Cell Microbiol.

[b16] Handa Y, Suzuki M, Ohya K, Iwai H, Ishijima N, Koleske AJ (2007). *Shigella* IpgB1 promotes bacterial entry through the ELMO-Dock180 machinery. Nat Cell Biol.

[b17] Hayashi T, Makino K, Ohnishi M, Kurokawa K, Ishii K, Yokoyama K (2001). Complete genome sequence of enterohemorrhagic *Escherichia coli* O157:H7 and genomic comparison with a laboratory strain K-12. DNA Res.

[b18] Imamura H, Takaishi K, Nakano K, Kodama A, Oishi H, Shiozaki H (1998). Rho and Rab small G proteins coordinately reorganize stress fibers and focal adhesions in MDCK cells. Mol Biol Cell.

[b19] Jaffe AB, Hall A (2005). Rho GTPases: biochemistry and biology. Ann Rev Cell Dev Biol.

[b20] Kakudo S, Kikuchi N, Kitadokoro K, Fujiwara T, Nakamura E, Okamoto H (1992). Purification, characterization, cloning, and expression of a glutamic acid-specific protease from *Bacillus licheniformis* ATCC 14580. J Biol Chem.

[b21] Kaper JB, Nataro JP, Mobley HL (2004). Pathogenic *Escherichia coli*. Nat Rev Microbiol.

[b22] Kenny B, Jepson M (2000). Targeting of an enteropathogenic *Escherichia coli* (EPEC) effector protein to host mitochondria. Cell Microbiol.

[b23] Kenny B, DeVinney R, Stein M, Reinscheid DJ, Frey EA, Finlay BB (1997). Enteropathogenic *E. coli* (EPEC) transfers its receptor for intimate adherence into mammalian cells. Cell.

[b24] Kenny B, Ellis S, Leard AD, Warawa J, Mellor H, Jepson MA (2002). Co-ordinate regulation of distinct host cell signalling pathways by multifunctional enteropathogenic *Escherichia coli* effector molecules. Mol Microbiol.

[b25] Knutton S, Lloyd DR, McNeish AS (1987). Adhesion of enteropathogenic *Escherichia coli* to human intestinal enterocytes and cultured human intestinal mucosa. Infect Immun.

[b26] Levine MM, Bergquist EJ, Nalin DR, Waterman DH, Hornick RB, Young CR, Sotman S (1978). *Escherichia coli* that cause diarrhoea but do not produce heat-labile or heat-stable enterotoxins and are non-invasive. Lancet.

[b27] Maekawa M, Ishizaki T, Boku S, Watanabe N, Fujita A, Iwamatsu A (1999). Signaling from Rho to the actin cytoskeleton through protein kinases ROCK and LIM-kinase. Science.

[b28] Marchès O, Covarelli V, Dahan S, Cougoule C, Bhatta P, Frankel G, Caron E EspJ of enteropathogenic and enterohaemorragic *Escherichia coli* inhibits opsono-phagocytosis. Cell Microbiol.

[b29] Matsuzawa T, Kuwae A, Yoshida S, Sasakawa C, Abe A (2004). Enteropathogenic *Escherichia coli* activates the RhoA signaling pathway via the stimulation of GEF-H1. EMBO J.

[b30] Ohya K, Handa Y, Ogawa M, Suzuki M, Sasakawa C (2005). IpgB1 is a novel Shigella effector protein involved in bacterial invasion of host cells. Its activity to promote membrane ruffling via Rac1 and Cdc42 activation. J Biol Chem.

[b31] Pelletier S, Julien C, Popoff MR, Lamarche-Vane N, Meloche S (2005). Cyclic AMP induces morphological changes of vascular smooth muscle cells by inhibiting a Rac-dependent signaling pathway. J Cell Physiol.

[b32] Riley LW, Junio LN, Libaek LB, Schoolnik GK (1987). Plasmid-encoded expression of lipopolysaccharide O-antigenic polysaccharide in enteropathogenic *Escherichia coli*. Infect Immun.

[b33] Sander EE, ten Klooster JP, van Delft S, van der Kammen RA, Collard JG (1999). Rac downregulates Rho activity: reciprocal balance between both GTPases determines cellular morphology and migratory behavior. J Cell Biol.

[b34] Schlosser-Silverman E, Elgrably-Weiss M, Rosenshine I, Kohen R, Altuvia S (2000). Characterization of *Escherichia coli* DNA lesions generated within J774 macrophages. J Bacteriol.

[b35] Shaw RK, Smollett K, Cleary J, Garmendia J, Straatman-Iwanowska A, Frankel G, Knutton S (2005). Enteropathogenic *E. coli* type III effectors EspG and EspG2 disrupt the microtubule network of intestinal epithelial cells. Infect Immun.

[b36] Simpson N, Shaw R, Mundy R, Crepin V, FitzGerald AJ, Cummings N (2006). The enteropathogenic *E. coli* type III secretion system effector Map binds EBP50/NHERF1: implication for cell signalling and diarrhoea. Mol Microbiol.

[b37] Tobe T, Tatsuno I, Katayama E, Wu CY, Schoolnik GK, Sasakawa C (1999). A novel chromosomal locus of enteropathogenic *Escherichia coli* (EPEC), which encodes a bfpT-regulated chaperone-like protein, TrcA, involved in microcolony formation by EPEC. Mol Microbiol.

[b38] Tobe T, Beatson SA, Taniguchi H, Abe H, Bailey CM, Fivian A (2006). Extensive repertoire of type III secretion effectors in *Escherichia coli* O157 and the role of lambdoid phages in their dissemination. Proc Natl Acad Sci USA.

[b39] Wiles S, Clare S, Harker J, Huett A, Young D, Dougan G (2004). Organ-specificity, colonization and clearance dynamics *in vivo* following oral challenges with the murine pathogen *Citrobacter rodentium*. Cell Microbiol.

[b40] Wong KW, Isberg RR (2005). *Yersinia pseudotuberculosis* spatially controls activation and misregulation of host cell Rac1. PloS Pathog.

[b41] Yamaguchi H, Miwa Y, Kasa M, Kitano K, Amano M, Kaibuchi K, Hakoshima T (2006). Structural basis for induced-fit binding of Rho-kinase to the inhibitor Y-27632. J Biochem.

[b42] Ziegler WH, Liddington RC, Critchley DR (2006). The structure and regulation of vinculin. Trends Cell Biol.

